# A competitive scheme for storing sparse representation of X-Ray medical images

**DOI:** 10.1371/journal.pone.0201455

**Published:** 2018-08-16

**Authors:** Laura Rebollo-Neira

**Affiliations:** Mathematics Department, Aston University, B4 7ET Birmingham, United Kingdom; University of Queensland, AUSTRALIA

## Abstract

A competitive scheme for economic storage of the informational content of an X-Ray image, as it can be used for further processing, is presented. It is demonstrated that sparse representation of that type of data can be encapsulated in a small file without affecting the quality of the recovered image. The proposed representation, which is inscribed within the context of data reduction, provides a format for saving the image information in a way that could assist methodologies for analysis and classification. The competitiveness of the resulting file is compared against the compression standards JPEG and JPEG2000.

## 1 Introduction

Sparse image representation refers to particular techniques for data reduction. Rather than representing the image informational content by the intensity of the pixels, the image is transformed with the aim of reducing the number of data points for reproducing the equivalent information. Lessening the cardinality of medical data is crucial for remote diagnosis and treatments [1–4]. The interest for this matter in the area of medical technology has recently been invigorated by the prospect of earlier disease detection, using neural networks and deep learning methodologies for automatic analysis of X-Ray plates [5–7].

In recent work [8] we have demonstrated that sparse representation, obtained by a large dictionary and greedy algorithms, renders high quality approximation of X-Ray images. The framework was proven to produce approximations which are far more sparser than those arising from traditional transformations such as the Cosine and Wavelet Transforms. Nevertheless, for the approximation to be useful within the context of automatic health care systems and remote reporting, it is necessary to ensure that the high levels of the achieved sparsity are not affected by saving the reduced data in a small file. This paper follows on the study in [8] by presenting a simple scheme to store the sparse approximation of an X-Ray medical image in a file of competitive size with respect to the most commonly used formats, namely JPEG and JPEG2000 (JPEG2).

Although in general X-Ray images are sparser if the approximation is performed in the wavelet domain, for analysis purposes the approximation in the pixel intensity domain may be needed. Hence, we consider here approximations in both domains. It is pertinent to stress that the aim of this work is not to propose yet one more method for image compression, but a method for economic storage of the informational content of an X-Ray image as it can be used for further processing [7, 9–15]. The comparison of the resulting file size, against those produced by JPEG and JPEG2, is carried out with the purpose of illustrating the effectiveness of the proposed scheme.

## 2 Sparse image representation

Throughout the paper R stands for the set of real numbers. Lower boldface letters are used to represent vectors and upper boldface letters to represent matrices. Standard mathematical fonts indicate their corresponding components, e.g., $d\in R^{N}$ is a vector of components *d*(*i*), *i* = 1, …, *N* and $I\in R^{N_{x}\times N_{y}}$ a matrix of elements *I*(*i*, *j*), *i* = 1, …, *N*_*x*_, *j* = 1, …, *N*_*y*_.

Suppose that an image, given as a 2D array $I\in R^{N_{x}\times N_{y}}$ of intensity values, is to be approximated by the linear decomposition

$$\begin{array}{c} I^{k}=\sum_{n=1}^{k}c\left(n\right)D_{\ell_{n}}, \end{array}$$ (1)

where each *c*(*n*) is a scalar and each $D_{\ell_{n}}$ is an element of $R^{N_{x}\times N_{y}}$ normalized to unity, called an ‘atom’, to be selected from a redundant set, $D={\left\{D_{n}\right\}}_{n=1}^{M}$, called a ‘dictionary’. The selection of the atoms $D_{\ell_{n}}$ in (1) is carried out according to an optimality criterion.

The goal of a sparse representation is to produce an approximation **I**^*k*^, of an image **I**, using as few atoms as possible. The mathematical methods for performing the task are either based on the minimization of the *l*_1_-norm [16, 17] or are greedy strategies for stepwise selection of atoms from the dictionary [18, 19]. When using large dictionaries, the latter are especially suited for practical applications. We focus here on the greedy algorithm known as Orthogonal Matching Pursuit (OMP), which is very effective at furnishing sparse solutions. In spite of the fact that the method is computationally intensive, the implementation described in the next section is very efficient in terms of processing time.

### 2.1 Orthogonal Matching Pursuit in 2D

The OMP method was introduced in [19] and has been implemented by a number of different algorithms. We describe a particular implementation for 2D, henceforth referred to as OMP2D. The dictionary is restricted to be separable, i.e. a 2D dictionary D which corresponds to the tensor product of two 1D dictionaries $D=D^{x}⊗D^{y}$. Our implementation of OMP2D is based on adaptive biorthogonalization and Gram-Schmidt orthogonalization procedures, as proposed in [20] for the one dimensional case. For the convenience of the interested researcher we include the description of its generalization to separable 2D dictionaries, as it is implemented by the MATLAB and C++ MEX functions we have made available.

Given a gray level intensity image, $I\in R^{N_{x}\times N_{y}}$, and two 1D dictionaries $D^{x}={\left\{d_{n}^{x}\in R^{N_{x}}\right\}}_{n=1}^{M_{x}}$ and $D^{y}={\left\{d_{m}^{y}\in R^{N_{y}}\right\}}_{m=1}^{M_{y}}$, we approximate the array $I\in R^{N_{x}\times N_{y}}$ by the atomic decomposition of the form:

$$\begin{array}{c} I^{k}=\sum_{n=1}^{k}c_{n}A_{n}=\sum_{n=1}^{k}c_{n}d_{\ell_{n}^{x}}^{x}{\left(d_{\ell_{n}^{y}}^{y}\right)}^{T}, \end{array}$$ (2)

where ${\left(d_{\ell_{n}^{y}}^{y}\right)}^{T}$ indicates the transpose of the column vector $d_{\ell_{n}^{y}}^{y}$. The OMP2D approach determines the atoms $A_{n}=d_{\ell_{n}^{x}}^{x}{\left(d_{\ell_{n}^{y}}^{y}\right)}^{T}$ in (2) as follows:

On setting *k* = 0 and **R**^0^ = **I** at iteration *k* + 1 the algorithm selects the elements $d_{\ell_{k+1}^{x}}^{x}\in D^{x}$ and $d_{\ell_{k+1}^{y}}^{y}\in D^{y}$ corresponding to the indices obtained as

$$\begin{array}{c} \begin{array}{cc} l_{k+1}^{x},l_{k+1}^{y} & =\underset{\begin{array}{l} n=1,\ldots ,M_{x}\\ m=1,\ldots ,M_{y} \end{array}}{\text{arg}\;\text{max}}\left|{\left(d_{n}^{x}\right)}^{T}R^{k}d_{m}^{y}\right|,\\ \text{with} & \\ R^{k} & =I-\sum_{n=1}^{k}c\left(n\right)d_{l_{n}^{x}}^{x}{\left(d_{l_{n}^{y}}^{y}\right)}^{T}. \end{array} \end{array}$$ (3)

The coefficients ${\left\{c\left(n\right)\right\}}_{n=1}^{k}$ in (3) are such that ‖**R**^*k*^‖_*F*_ is minimum (‖⋅‖_F_ being the Frobenius norm induced by the Frobenius inner product 〈⋅, ⋅〉_F_). This is guaranteed by calculating the coefficients as

$$\begin{array}{c} c_{n}={\left\langle B_{n}^{k},I\right\rangle }_{F},\;n=1,\ldots ,k, \end{array}$$ (4)

where the matrices ${\left\{B_{n}^{k}\right\}}_{n=1}^{k}$ are recursively constructed, at each iteration, to account for each newly selected atom. Starting from $B_{1}^{1}=W_{1}=A_{1}=d_{\ell_{1}^{x}}^{x}{\left(d_{\ell_{1}^{y}}^{y}\right)}^{T}$ the set of matrices is upgraded and updated through the formula:

$$\begin{array}{c} \begin{array}{cc} B_{n}^{k+1} & =B_{n}^{k}-B_{k+1}^{k+1}{\left\langle A_{k+1},B_{n}^{k}\right\rangle }_{F},\;n=1,\ldots ,k,\\ \;\;\;\text{where}\\ B_{k+1}^{k+1} & =W_{k+1}/{∥W_{k+1}∥}_{F}^{2},\;\;\text{with}\\ W_{k+1} & =A_{k+1}-\sum_{n=1}^{k}\frac{W_{n}}{∥W_{n}{∥}_{F}^{2}}{\left\langle W_{n},A_{k+1}\right\rangle }_{F}. \end{array} \end{array}$$ (5)

For numerical accuracy of the orthogonal set ${\left\{W_{n}\right\}}_{n=1}^{k+1}$ at least one re-orthogonalization step is usually needed. It requires the recalculation of the element **W**_*k*+1_ as

$$\begin{array}{c} W_{k+1}\leftarrow W_{k+1}-\sum_{n=1}^{k}\frac{W_{n}}{∥W_{n}{∥}_{F}^{2}}.{\left\langle W_{n},W_{k+1}\right\rangle }_{F}. \end{array}$$ (6)

The algorithm iterates until for a given parameter *ρ* the stopping criterion $||I-I^{k}{\left|\right|}_{F}^{2}<\rho$ is met.

### 2.2 Approximation by partitioning

The OMP2D approach described above is to be applied on an image partition. This implies the division of the image into small disjoint blocks **I**_*q*_, *q* = 1, …, *Q*, which without loss of generality we assume to be square of dimension *N*_*b*_ × *N*_*b*_. Denoting each element of the image partition by **I**_*q*_, these are approximated, independently of each other, to produce the approximations $I_{q}^{k_{q}}$, *q* = 1, …, *Q*, of the form (2). The superscript *k*_*q*_ indicates the number of atoms intervening in the decomposition of the block *q*. As already mentioned, in spite of the fact that the approximation of most X-Ray images is significantly sparser if performed in the wavelet domain, results in the pixel intensity domain may be required for particular applications.

**Fig 1:**
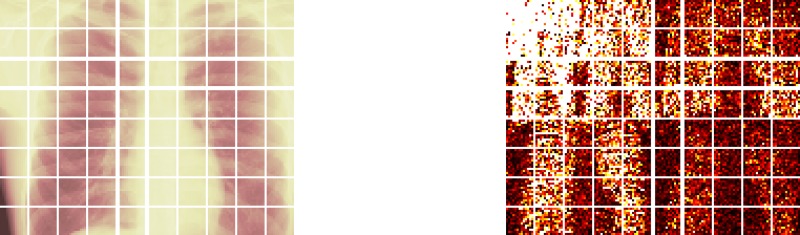
Portion of a chest image. Illustration of an X-Ray image partition in the pixel intensity domain (left graph) and in the wavelet domain (right graph). The image size is 256 × 320 pixels and the partition is illustrated by blocks of 32 × 32 pixels. The colours are added for visual effect.

The approximation in the wavelet domain involves a transformation, via a Wavelet Transform, which converts the image into the array to be approximated. The inverse transformation is taken after the approximation is concluded. Fig 1 provides graphical illustrations of image partitions in the pixel intensity and the wavelet domain.

**Remark 1:** The OMP2D algorithm described in Sec. 2.1 is very effective up to some block size. While the actual size depends on the sparsity of the image, previous studies indicate that in general for block sizes greater than 24 × 24 an alternative implementation is advisable. The alternative implementation, called Self Projected Matching Pursuit (SPMP2D) [8, 21] is not only dedicated to tackling large dimensional problems, but also potentially suitable for implementation in Graphic Processing Unit (GPU) programming. However, because for X-Ray medical images a partition into blocks of size 16 × 16 is a good compromise between sparsity and processing time, we have focussed on the implementation of OMP2D as given in Sec. 2.1. Other possibilities for approximating a partition are discussed in [8, 22].

### 2.3 Mixed dictionaries

The available literature in relation to the construction of dictionaries for image representation is mainly concerned with methodologies for learning atoms from training data [23–28]. Those methodologies are not designed for learning the types of dictionaries of our interest though. We restrict the dictionary to be separable, in order to reduce the computational burden and memory requirements. In previous works [8, 21, 29] we have demonstrated that very large separable dictionaries, which are easy to construct, render high levels of sparsity. Such dictionaries are not specific to a particular class of images. A discrimination is only made to take into account whether the approximation is carried out in the pixel intensity or in the wavelet domain. In each domain we use mixed dictionaries which are similar in nature, but not equal. They have the trigonometric dictionaries $D_{C}^{x}$ and $D_{S}^{x}$, defined below, as common components.

$$\begin{array}{c} \begin{array}{cc} D_{C}^{x} & =\;{\left\{w_{c}\left(n\right)\cos\frac{\pi (2i-1)(n-1)}{2M},i=1,\ldots ,N\right\}}_{n=1}^{M}\\ D_{S}^{x} & =\;{\left\{w_{s}\left(n\right)\sin\frac{\pi (2i-1)(n)}{2M},i=1,\ldots ,N\right\}}_{n=1}^{M}, \end{array} \end{array}$$

where *w*_*c*_(*n*) and *w*_*s*_(*n*), *n* = 1, …, *M* are normalization factors, and usually *M* = 2*N*.

**Fig 2:**
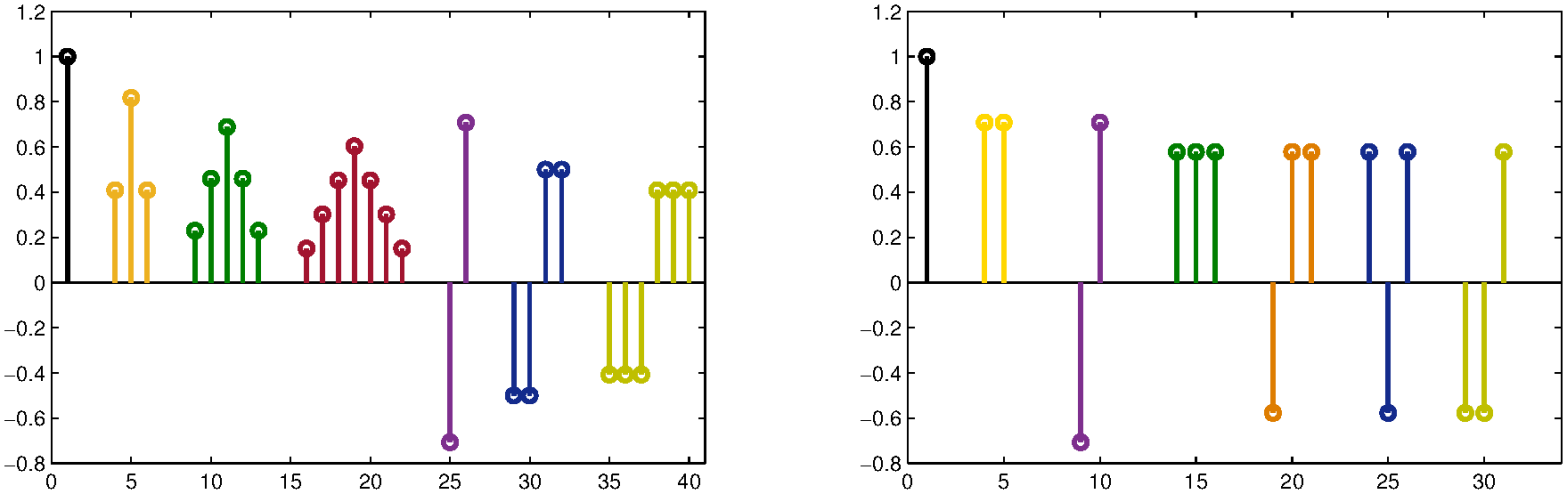
Prototype atoms, which generate by translation the dictionaries $D_{\text{Lp}}^{x}$ (left) and $D_{\text{Lw}}^{x}$ (right).

For approximations in the wavelet domain we add the dictionary $D_{\text{Lw}}^{x}$, as proposed in [8], which is built by translation of the prototype atoms in the right graph of Fig 2. The mixed dictionary $D_{\text{wd}}^{x}$, to be used only in the wavelet domain, is formed as $D_{\text{wd}}^{x}=D_{C}^{x}\cup D_{S}^{x}\cup D_{\text{Lw}}^{x}$ and $D_{\text{wd}}^{y}=D_{\text{wd}}^{x}$.

For approximations in the pixel intensity domain we add the dictionary, $D_{\text{Lp}}^{x}$, which is built by translation of the prototype atoms in the left graph of Fig 2. In this case the mixed dictionary $D_{\text{pd}}^{x}$ is formed as $D_{\text{pd}}^{x}=D_{C}^{x}\cup D_{S}^{x}\cup D_{\text{Lp}}^{x}$ and $D_{\text{pd}}^{y}=D_{\text{pd}}^{x}$.

The corresponding 2D dictionaries $D_{\text{wd}}=D_{\text{wd}}^{x}⊗D_{\text{wd}}^{y}$ and $D_{\text{pd}}=D_{\text{pd}}^{x}⊗D_{\text{pd}}^{y}$ are very large, but not needed as such. All calculations are carried out using the 1D dictionaries.

## 3 Coding strategy

For the sparse representation of an X-Ray image to be useful within the current trend of medical technology developments it should be suitable to be encapsulated in a small file. Accordingly, the coefficients of the atomic decompositions need to be converted into integer numbers. This operation is known as quantization. We adopt a simple and commonly used uniform quantization technique. For *q* = 1, …, *Q* the absolute value coefficients |*c*_*q*_(*n*)|, *n* = 1, …, *k*_*q*_ are converted to integers as follows:

$$\begin{array}{c} c_{q}^{\Delta }\left(n\right)=\{\begin{array}{cc} \lceil \frac{|c_{q}\left(n\right)|-\theta }{\Delta }\rceil , & \text{if}\;|c_{q}\left(n\right)|\geq \theta \\ & 0\;\text{otherwise}, \end{array} \end{array}$$ (7)

where ⌈*x*⌉ indicates the smallest integer number greater than or equal to *x*, Δ is the quantization parameter, and *θ* the threshold to disregard coefficients of small magnitude. The signs of the coefficient are encoded separately, as a vector **s**_*q*_, using a binary alphabet.

In order to store the information about the particular atoms present in the approximation of each block, we proceed as follows: Firstly each pair of indices $\left(\ell_{n}^{x,q},\ell_{n}^{y,q}\right)$ corresponding to the atoms in the decompositions of the block **I**_*q*_ is mapped into a single index *o*_*q*_(*n*). Then the set *o*_*q*_(1), …, *o*_*q*_(*k*_*q*_) is sorted in ascending order $o_{q}\left(n\right)\to {\tilde{o}}_{q}\left(n\right),\;n=1,\ldots ,k_{q}$. This guarantees that, for each *q*-value, ${\tilde{o}}_{q}\left(i\right)<{\tilde{o}}_{q}\left(i+1\right),\;i=1,\ldots ,k_{q}-1$. The order of the indices induces an order in the unsigned coefficients, $c_{q}^{\Delta }\to {\tilde{c}}_{q}^{\Delta }$ and in the corresponding signs $s_{q}\to {\tilde{s}}_{q}$. The advantage introduced by the ascending order of the indices is that they can be stored as smaller positive numbers, by taking differences between two consecutive values. Certainly by defining $\delta_{q}\left(n\right)={\tilde{o}}_{q}\left(n\right)-{\tilde{o}}_{q}\left(n-1\right),\;n=2,\ldots ,k_{q}$ the string ${\tilde{o}}_{q}\left(1\right),\delta_{q}\left(2\right),\ldots ,\delta_{q}\left(k_{q}\right)$ stores the indices for the block *q* with unique recovery. The number 0 is then used to separate the strings corresponding to different blocks.

$$\begin{array}{c} st_{\text{ind}}\;=\;\left[{\tilde{o}}_{1}\left(1\right),\delta_{1}\left(2\right),\ldots ,\delta_{1}\left(k_{1}\right),0,{\tilde{o}}_{2}\left(1\right),\delta_{2}\left(2\right),\ldots ,\delta_{2}\left(k_{2}\right),0,\cdots ,{\tilde{o}}_{k_{Q}}\left(1\right),\delta_{k_{Q}}\left(2\right)\ldots ,\delta_{k_{Q}}\left(k_{Q}\right)\right]. \end{array}$$ (8)

The quantized magnitude of the re-ordered coefficients are concatenated in the strings *st*_cf_ as follows:

$$\begin{array}{c} st_{\text{cf}}=\left[{\tilde{c}}_{1}^{\Delta }\left(1\right),\ldots ,{\tilde{c}}_{1}^{\Delta }\left(k_{1}\right),{\tilde{c}}_{2}^{\Delta }\left(1\right),\ldots ,{\tilde{c}}_{2}^{\Delta }\left(k_{2}\right),\cdots ,{\tilde{c}}_{k_{Q}}^{\Delta }\left(1\right),\ldots ,{\tilde{c}}_{k_{Q}}^{\Delta }\left(k_{Q}\right)\right]. \end{array}$$ (9)

Using 0 if the sign is positive and 1 if it is negative, the signs of the coefficients are placed in the string, *st*_sg_ as

$$\begin{array}{c} st_{\text{sg}}=\left[{\tilde{s}}_{1}\left(1\right),\ldots ,{\tilde{s}}_{1}\left(k_{1}\right),{\tilde{s}}_{2}\left(1\right),\ldots ,{\tilde{s}}_{2}\left(k_{2}\right),\cdots ,{\tilde{s}}_{k_{Q}}\left(1\right),\ldots ,{\tilde{s}}_{k_{Q}}\left(k_{Q}\right)\right]. \end{array}$$ (10)

The next encoding/decoding scheme summarizes the above described procedure.

**Encoding**
- Given an image partition $I_{q}\in R^{N_{b}\times N_{b}},\;q=1,\ldots ,Q$ use OMP2D (or other method) to approximate each element of the partition by the atomic decomposition:
  $$\begin{array}{c} I_{q}^{k_{q}}=\sum_{n=1}^{k_{q}}c_{q}\left(n\right)d_{\ell_{n}^{x,q}}^{x}{\left(d_{\ell_{n}^{y,q}}^{y}\right)}^{T}. \end{array}$$ (11)
- The approximation is carried out on each block, independently of the others, until the stopping criterion is reached.
- For each *q*, quantize as in (7) the magnitude of the coefficients in the decomposition (11) to obtain $c_{q}^{\Delta }\left(n\right),\;n=1,\ldots ,k_{q}$. Store the signs of the non-zero coefficient as components of a vector **s**_*q*_.
- For each *q*, map the pair of indices $\left(\ell_{n}^{x,q},\ell_{n}^{y,q}\right),\;n=1,\ldots ,k_{q}$ in (11) into a single index *o*_*q*_(*n*), *n* = 1, …, *k*_*q*_ and sort these numbers in ascending order to have the re-ordered sets: ${\tilde{o}}_{q}\left(1\right)\ldots ,{\tilde{o}}_{q}\left(k_{q}\right)$; ${\tilde{c}}_{q}^{\Delta }\left(1\right),\ldots ,{\tilde{c}}_{q}^{\Delta }\left(k_{q}\right)$ and ${\tilde{s}}_{q}\left(1\right),\ldots ,{\tilde{s}}_{q}\left(k_{q}\right)$ to create the strings: *st*_ind_, as in (8), and *st*_cf_, and *st*_sg_ as in (9) and (10) respectively.

Let’s recall the content of the file encoded by the above steps:

*st*_ind_ contains the difference of indices corresponding to the atoms in the approximation of each of the blocks in the image partition.
*st*_cf_ contains the magnitude of the corresponding coefficients (quantized to integer numbers).
*st*_sg_ contains the signs of the coefficients in binary format. The quantization parameter Δ also needs to be stored in the file. We fix *θ* = 1.3Δ for all the images.

**Decoding**
- Recover the indices from their difference. This operation also gives the information about the number of coefficients in each block.
- Read the quantized unsigned coefficients from the string *st*_cf_ and transform them into real numbers as $|{\tilde{c}}_{q}^{r}\left(n\right)|=\Delta {\tilde{c}}_{q}^{\Delta }\left(n\right)+\left(\theta -\Delta /2\right)$. Read the corresponding signs from the string *st*_sg_.
- Recover the approximated partition, for each block, through the liner combination
  $$\begin{array}{c} I_{q}^{r,k_{q}}=\sum_{n=1}^{k_{q}}{\tilde{s}}_{q}\left(n\right)\left|{\tilde{c}}_{q}^{r}\left(n\right)\right|d_{{\tilde{\ell }}_{n}^{x,q}}^{x}{\left(d_{{\tilde{\ell }}_{n}^{y,q}}^{y}\right)}^{T}. \end{array}$$
- Assemble the recovered image as
  $$\begin{array}{c} I^{r,K}={\hat{J}}_{q=1}^{Q}I_{q}^{r,k_{q}}, \end{array}$$
  where the $\hat{J}$ indicates the operation for joining the blocks to restore the image.

**Note:** The MATLAB scripts for implementing the scheme and reproducing the results presented in the next section have been made available on [30].

### 3.1 Evaluation metrics

The quality of the approximation is quantified by the Mean Structural SIMilarity (MSSIM) index [31, 32] and the classical Peak Signal-to-Noise Ratio (PSNR), calculated as

$$\begin{array}{c} \text{PSNR}=10\log_{10}\;(\frac{{\left(2^{8}-1\right)}^{2}}{\text{MSE}}),\;\text{with}\;\text{MSE}=\frac{∥I-I^{r,K}{∥}_{F}^{2}}{N_{x}N_{y}}. \end{array}$$ (12)

The required MSSIM is set to be MSSIM ≥ 0.997. This limit guarantees that the approximation is indistinguishable from the image, in the original size (it should be noticed that the MSSIM between an image and itself is 1). For the comparison with standard formats all the PSNRs are fixed as values for which JPEG and JPEG2 produce the required MSSIM. For producing a requested PSNR with the sparse representation approach we proceed as follows: the approximation routine is set to yield a slightly larger value of PSNR and the required one is then obtained by tuning the quantization parameter Δ.

As a measure of sparsity we use the Sparsity Ratio, which is defined as [33]:

$$\begin{array}{c} \text{SR}=\frac{\text{Number}\;\text{of}\;\text{pixels}\;\text{in}\;\text{the}\;\text{image}}{\text{Number}\;\text{of}\;\text{coefficients}\;\text{in}\;\text{the}\;\text{representation}}. \end{array}$$ (13)

Accordingly, the sparsity of a representation is manifested as a high value of SR.

In addition to the SR, which is a global measure of sparsity, a meaningful description of the variation of the image content throughout the partition is rendered by the local sparsity ratio, which is given as

$$\begin{array}{c} sr\left(q\right)=\frac{N_{b}^{2}}{k_{q}},\;q=1,\ldots ,Q, \end{array}$$ (14)

where *k*_*q*_ is the number of coefficients in the decomposition of the *q*-block and $N_{b}^{2}$ is the number of pixels in the block.

### 3.2 Data sets

We illustrate the effectiveness of the proposed encoding scheme by storing the outputs of high quality sparse approximation of two data sets: 1) the Lukas 2D 8 bit medical image corpus, available on [34] and 2) the sample of 25 X-ray chest images taken randomly from the National Institute of Health (NIH) dataset available on [35].

**Fig 3:**
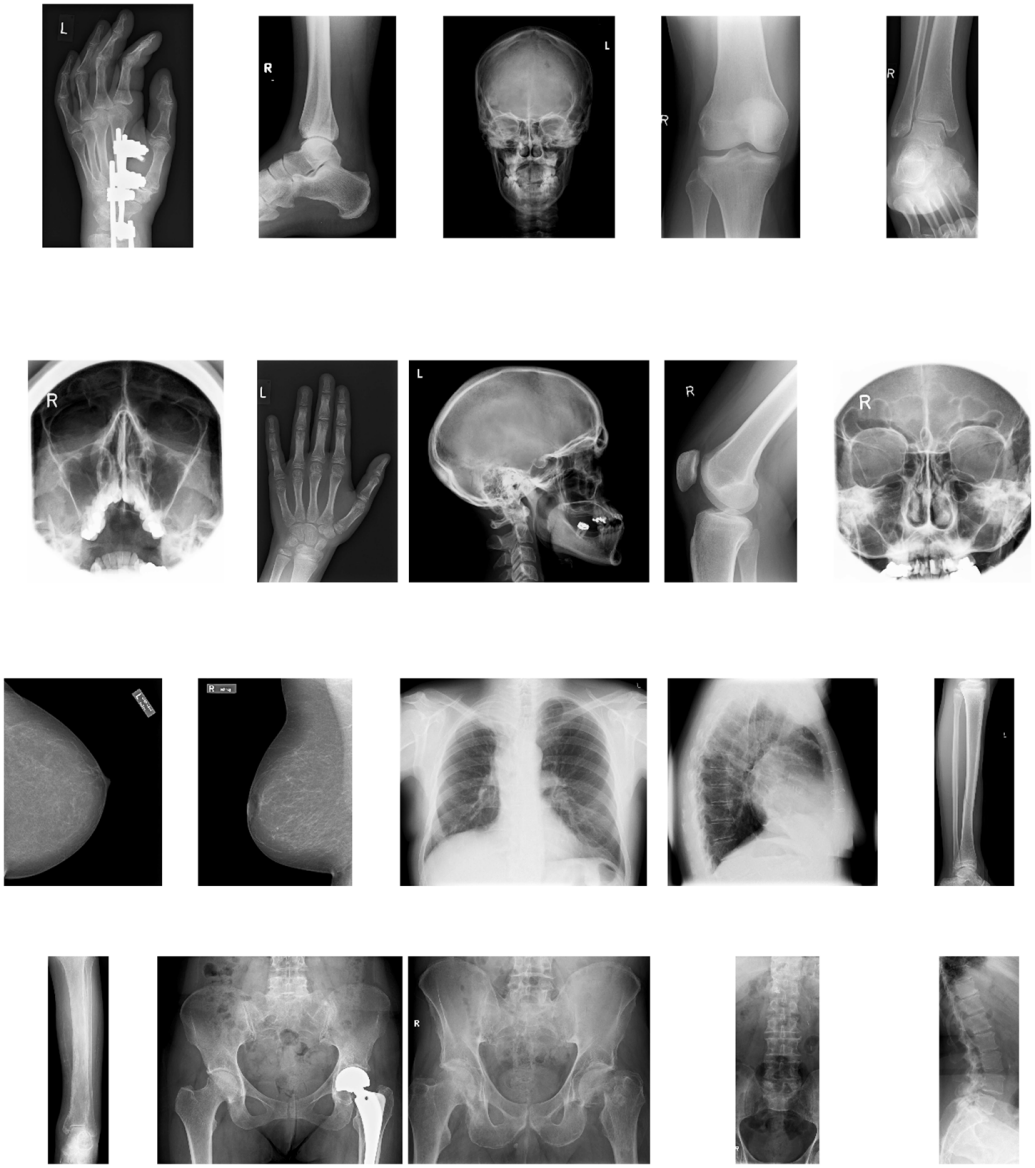
Lukas data set. The 20 images in the Lukas Corpus [34] listed in Table 1. First row: Hand_1_, Foot_0_, Head_0_, Knee_1_, Foot_1_. Second row: Sinus_0_, Hand_0_, Head_1_, Knee_0_, Sinus_1_. Third row: Breast_0_, Breast_1_, Thorax_0_, Thorax_1_, Leg_0_. Fourth row: Leg_1_, Pelvis_1_, Pelvis_0_, Spine_1_, Spine_0_. The average size of these images is 1943 × 1364 pixels.

The Lukas corpus consists of 20 images which cover different parts of the human body, as shown in Fig 3. The average size of the images in the set is 1943 × 1364 pixels.

The chest X-Ray images are available in the raster graphics file format portable network graphics (png). Information related to the NIH X-Ray collection is given in [36]. The 25 images used here have been placed on [30]. The sample consists of common views of the chest in radiology. The posteroanterior, anteroposterior, and lateral views, which are obtained by changing the relative orientation of the body and the direction of the X-Ray beam. The images in this set are of similar size and smaller than in the Lukas corpus. The average size is 496 × 512 pixels.

## 4 Results

The approximation of all the images in the Lukas corpus are performed in both the pixel intensity and the wavelet domain. The size of the blocks in the image partition is fixed taking into account previously reported results [8, 21], which indicate that 16 × 16 is a good trade-off between the resulting sparsity and the processing time. The information about the sizes of the corresponding files is given in bits per pixel (bpp) in the third and forth columns of Table 1. The results for JPEG and JPEG2 are placed in the fifth and sixth columns, respectively. The last two rows of the table are the mean value of standard deviation (std) of the corresponding columns.

**Table 1:** Comparison of size rate (in bpp) for the Lukas corpus, listed in the first column. The third column shows the bpp values corresponding to the sparse representation in the pixel domain (S_pd_). The forth column shows the corresponding results in the wavelet domain (S_wd_). The fifth and sixth columns are the bpp values for the formats JPEG and JPEG2, respectively. All the approaches render a MSSIM ≥ 0.997 and the values of PSNR listed in the second column.

Image	dB	S_pd_	S_wd_	JPEG	JPEG2
1 Hand_1_	48.1	0.443	0.286	0.436	0.234
2 Foot_0_	48.6	0.462	0.306	0.449	0.247
3 Head_0_	47.4	0.441	0.320	0.419	0.244
4 Knee_1_	48.0	0.488	0.316	0.485	0.286
5 Foot_1_	48.1	0.624	0.391	0.566	0.335
6 Sinus_0_	47.1	0.676	0.416	0.575	0.334
7 Hand_0_	48.8	0.612	0.424	0.586	0.371
8 Head_1_	46.4	0.599	0.452	0.546	0.346
9 Knee_0_	49.1	0.612	0.419	0.573	0.364
10 Sinus_1_	45.8	0.697	0.453	0.594	0.358
11 Breast_0_	44.3	0.519	0.465	0.605	0.393
12 Breast_1_	44.3	0.691	0.629	0.832	0.521
13 Thorax_0_	44.1	0.827	0.686	0.874	0.629
14 Thorax_1_	43.4	0.816	0.693	0.924	0.619
15 Leg_0_	48.9	1.000	0.867	1.152	0.759
16 Leg_1_	49.2	1.384	1.240	1.584	1.056
17 Pelvis_1_	44.3	1.596	1.418	1.828	1.248
18 Pelvis_0_	44.4	1.606	1.435	1.827	1.310
19 Spine_1_	47.0	2.131	1.922	2.463	1.630
20 Spine_0_	47.4	2.395	2.298	2.764	1.902
Mean Value	46.7	0.938	0.727	1.004	0.659
Std	1.9	0.573	0.521	0.707	0.500

As shown in Table 1, all the files corresponding to approximations in the wavelet domain (S_wd_) are smaller than those corresponding to approximations in the pixel intensity domain (S_pd_), and also smaller than the JPEG ones. On average the files with the sparse representation in the wavelet domain are 22% smaller than those with the representation in the pixel domain, and 27% smaller than the JPEG files. In the present form JPEG2 produces the smallest files (on average 9% smaller than the files with the representation in the wavelet domain). However, both JPEG and JPEG2 formats involve an entropy coding step, which is not included in our scheme. Instead, the outputs of our algorithm are stored in HDF5 format [37, 38]. In the provided software [30] this is implemented in a straightforward manner using the MATLAB function save. On the contrary, adding an entropy coding step to the software would increase the processing time.

**Fig 4:**
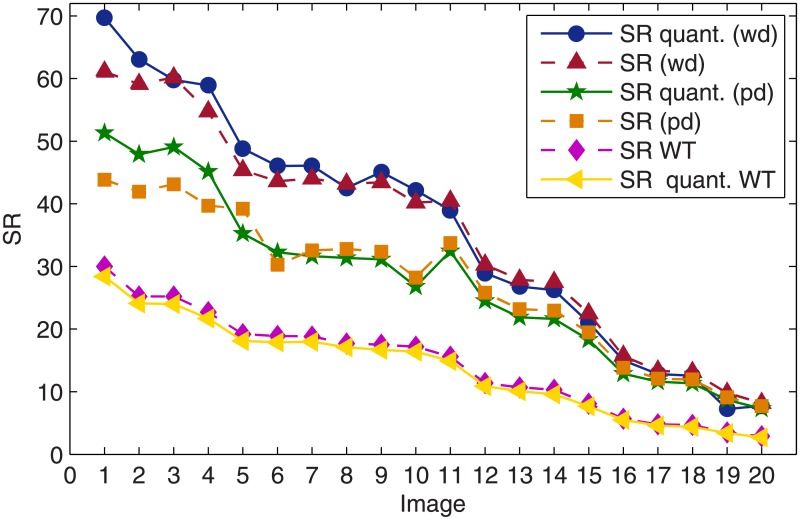
Comparison of the SRs, before and after quantization, corresponding to the dictionary approach in both the pixel intensity and the wavelet domain and to the wavelet approximation by nonlinear thresholding.

A very interesting feature of the numerical results is that the quantization process, intrinsic to the economic store of the coefficients in the image approximation does not reduce the sparsity. For the sake of comparison in addition to calculating the SRs obtained with the dictionary approach in both domains, before and after quantization, we have also calculated the corresponding SRs produced by nonlinear thresholding of the wavelet coefficients. The results are shown in Fig 4.

As already discussed, since the quantization of coefficients degrades quality, to achieve the required PSNR the approximation of the image has to be carry out up to a higher PSNR value. Nevertheless, because the quantization process maps some coefficients to zero, for the corpus of 20 images in this study, quantization does not affect sparsity. On the contrary, as can be observed in Fig 4, for the sparsest images (first 5 images in Table 1) sparsity actually benefits from quantization. It is also clear that for the images in the upper part of the table the SR in the wavelet domain is significantly larger than in the pixel intensity domain. However, the level of sparsity achieved by the dictionary approach is, in both domains, significantly higher than that achieved by nonlinear thresholding of the wavelet coefficients. If the dictionary approach operates in the wavelet domain, then after quantization the mean value gain in SR with respect to thresholding of the wavelet coefficients is 163%, with standard deviation of 17%. In the pixel intensity domain the corresponding gain is 113%, with standard deviation of 30%.

**Fig 5:**
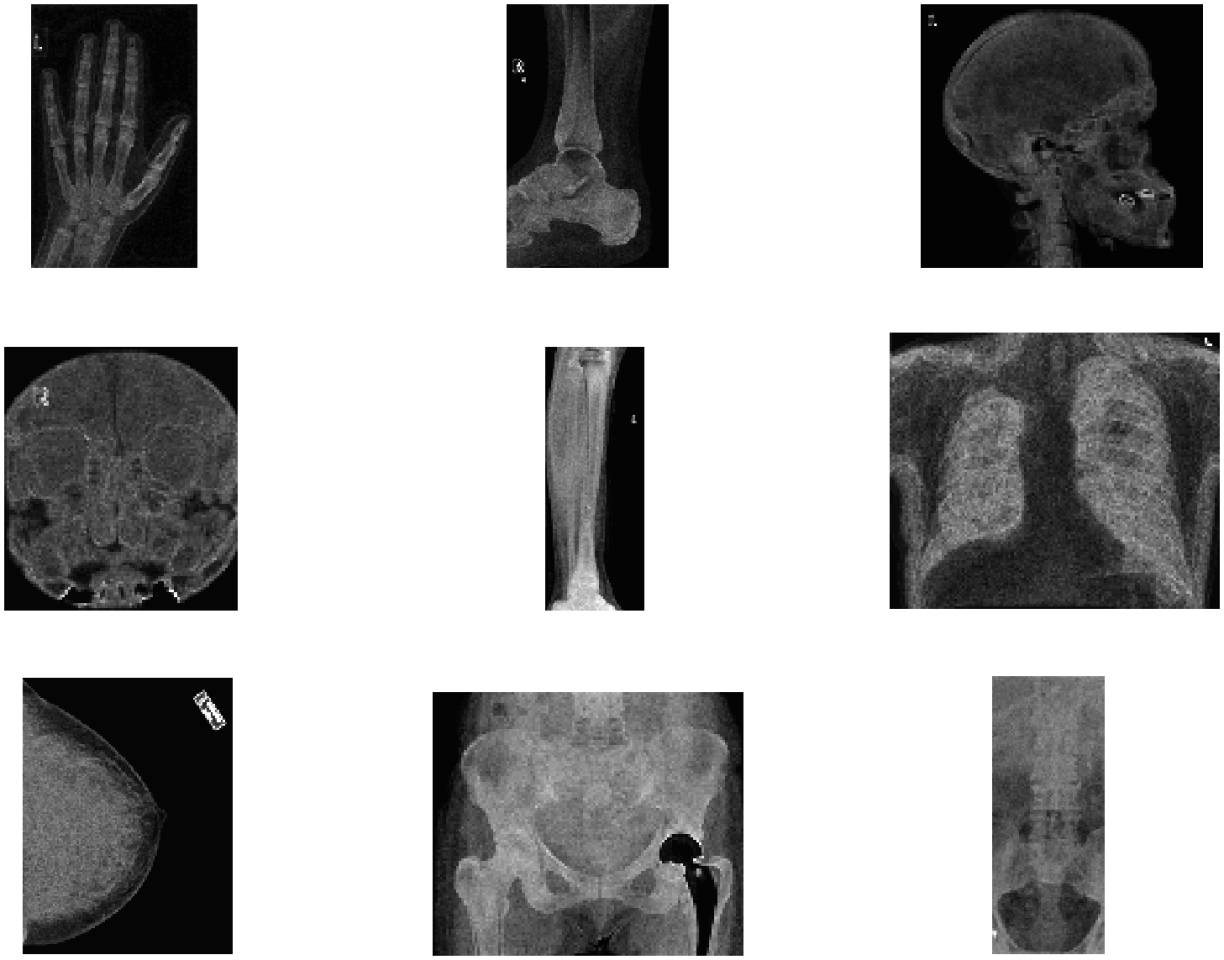
Digital summary of nine of the images in the Lukas corpus. The points in each graph are the inverse of the local sparsity ratio for nine of the images in Fig 3. The sizes of the graphs are (from top left to bottom right) 124 × 78, 140 × 86, 97 × 104, 99 × 88, 132 × 38, 107 × 128, 1138 × 105, 100 × 118 and 131 × 53 pixels.

It is worth mentioning that the local sparsity ratio (c.f. (14)) can be used to produce a digital summary of the images. Indeed, each of the graphs of Fig 5 depicts the inverse of the local sparsity ratio in the pixel domain, for nine of the images in the Lukas corpus. Each of the points in a graph represents the number of coefficients in the atomic decomposition of each block in the image partition. Hence, the number of points in each of the graphs of Fig 5 is equal to the number of blocks in the corresponding image partition. Note that showing the inverse of the local sparsity ratio implies that the brightest pixels correspond to the least sparse blocks. By comparing the graphs in Fig 5 with the images in Fig 3 it is clear that each of the graphs in Fig 5 corresponds to one of the image in the Lukas corpus: Hand_0_, Foot_1_, Head_1_, Sinus_1_, Leg_0_, Thorax_0_, Breast_0_, Pelvis_1_ and Spine_1_. This suggests that the digital summary of the images could be of assistance to classification and feature extraction techniques. For recent publications in that area of application see [39–45].

**Fig 6:**
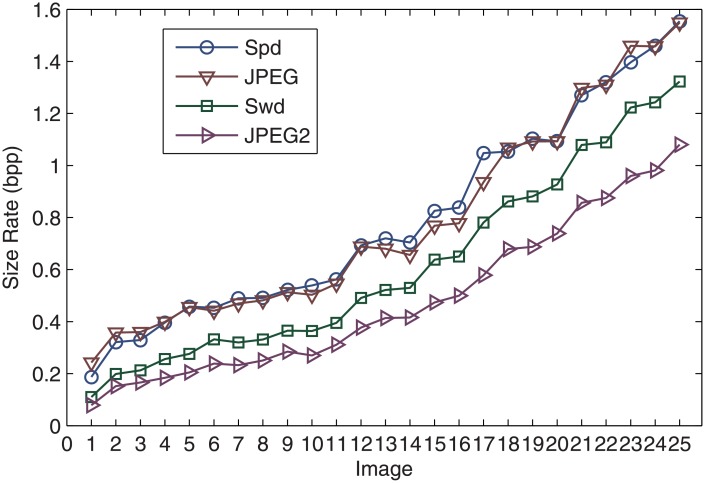
Comparison of the file size rate, in bpp, against the JPEG and JPEG2 formats for the 25 chest images. S_pd_ and S_wd_ correspond to the files containing the representation of the dictionary approach in the pixel and wavelet domains, respectively.

The approximation of the 25 chest X-Ray images is carried out to achieved a PSNR of 45dB for all the images. This guarantees a MSSIM of at least 0.99 for every image. The resulting size of the files lead to the same remarks as the values in the previous example displayed in Table 1. Indeed, as shown in Fig 6, the size rate produced by the dictionary approach in the pixel intensity domain (S_pd_) is competitive with the JPEG format for the same quality. The files produced in the wavelet domain (S_wd_) are smaller than the JPEG files but larger than the JPEG2 ones. However, let’s recall that the entropy coding step, which is part of the JPEG and JPEG2 compression standards, is not included in our scheme. This results in larger files but fast processing. Certainly, using MATLAB environment and a small notebook 2.9GHz dual core i7 3520M CPU and 4GB of memory, the average time for carrying out the approximation and creating the files of the 25 images with the dictionary approach is 1.7 s per image (with standard deviation 0.5). This time is the average of five independent runs in the time domain and another five independent runs in the wavelet domain.

In this case the sparsity of the image representation benefits from quantization even more than in the previous case: The dictionary approach in the pixel intensity domain yields a mean value SR ($\bar{\text{SR}}$) equal to 25.4 before quantization and $\bar{\text{SR}}=36.7$ and after quantization. In the wavelet domain $\bar{\text{SR}}=35.4$ before quantization, while $\bar{\text{SR}}=52.0$ after quantization.

## 5 Conclusions

A scheme for encapsulating the sparse representation of an X-Ray image in a small file has been proposed. The approach operates within the over-complete dictionary framework, which achieves a notable level of sparsity in the image representation. An interesting feature, emerging from the numerical results illustrating the approach, is that the process of devising the small file does not affect the sparsity of the representation. We consider this a very important outcome, because the central aim of the approach is to reduce, as much as possible, the cardinality of the data representing the informational content of the images. Additionally, the competitivity of the file size was compared against the standard formats JPEG and JPEG2. The MATLAB routines, for implementing all the steps of the process to reproduce the results in Table 1 and Fig 6, have been made available on a dedicated website [30].

As a final remark it should be mentioned that the quality of the image representation in the present study is very high. A lower value of MSSIM might be acceptable as diagnostically lossless representation of X-Ray images [32]. Within the proposed scheme the only effects of setting a lower value of MSSIM are: a) the approximation routine runs faster, since less atoms are chosen, and b) the storage file is of course smaller. The right value of MSSIM is to be set according to how the file is to be used whilst taking into account specialized opinions.
